# Comparison of Visual Field Fluctuation Between Myopic and Emmetropic Glaucoma Patients

**DOI:** 10.1155/joph/9948265

**Published:** 2025-09-21

**Authors:** Molly Barten, Louis B. Cantor

**Affiliations:** ^1^School of Medicine, Indiana University, Indianapolis, Indiana, USA; ^2^Department of Ophthalmology, Indiana University, Indianapolis, Indiana, USA

## Abstract

**Background:** Conflicting studies suggest that myopia may both increase and decrease the risk of developing glaucomatous visual field loss. To provide appropriate treatment, one must distinguish between visual field fluctuation, where variability occurs over days to weeks not due to pathological change, and true visual field progression.

**Objective:** A retrospective, observational clinical study tests the hypothesis that myopic glaucoma patients demonstrate more long-term visual field fluctuation than emmetropic glaucoma patients.

**Methods:** Four or more Humphrey visual field tests over several clinic visits were analyzed in 156 eyes. The visual field index (VFI) plot, mean deviation (MD), and threshold sensitivity on the glaucoma progression analysis were collected to quantify fluctuation. The sample had two groups: (1) emmetropic glaucoma eyes with a spherical equivalent refraction between +2 and −2 diopters and (2) high myopic glaucoma eyes with a spherical equivalent refraction ≤ −5 diopters. Two sample -tests and one-way analysis of variance (ANOVA) tests with random effect on subject using root mean square error of approximation (RMSEA) were used to compare fluctuation.

**Results:** RMSEA two sample -tests between 78 myopic and 78 emmetropic glaucoma eyes found that myopic glaucoma eyes demonstrated significant fluctuation for MD (*p* = 0.006) and VFI (*p* = 0.035) compared with emmetropic eyes. Although myopic eyes showed greater fluctuation in MD (1.504 ± 1.016) and VFI (0.037 ± 0.045) compared with emmetropic glaucoma eyes (MD = 1.125 ± 0.652) (VFI = 0.026 ± 0.019), threshold sensitivity analyses did not reach statistically significant differences (*p* = 0.054) between myopic (1.609 ± 1.022) and emmetropic glaucoma eyes (1.339 ± 0.687). One-way ANOVA tests found statistically significant MD and VFI fluctuation difference (MD = −0.29, *p* value = 0.01) (VFI = −0.30, *p* value = 0.03) but not threshold sensitivity fluctuation difference = −0.19 (*p* value = 0.11).

**Conclusion:** The results for MD and VFI, but not threshold sensitivity, are consistent with the hypothesis that myopic patients demonstrate more visual field fluctuation than emmetropic glaucoma patients.

## 1. Introduction

Glaucoma is the second leading cause of blindness with over 66 million people impacted worldwide and approximately 7 million people experiencing bilateral blindness [1]. Glaucoma is characterized by progressive atrophy of the optic nerve with an excavated appearance of the optic disc as well as visual field loss [2, 3]. The lamina cribrosa—where nerve fibers pass through the posterior sclera before exiting the eyes—is thought to be where the earliest glaucomatous damage occurs [4]. Glaucoma patients show a thinner lamina cribrosa compared with control patients [5].

Myopia, or nearsightedness, may have an important relationship with glaucoma. Myopia is very common with over half of the population in developed countries having the disease, and the percentage is expected to increase [6]. Studies have found conflicting evidence concerning myopia being a risk factor for glaucoma, with some studies showing that myopia has no effect on glaucoma risk [7] and others showing that myopia may increase the risk of glaucoma [8]. One study found that myopia increases the risk of glaucoma two to three times compared with patients without myopia [9]. There are several proposed mechanisms for myopia increasing the risk for glaucoma. Increased myopia severity is associated with increased optic disk tilt, longer axial length, and reduced sensitivity on visual field testing [10]. Another study has shown that the trabecular meshwork, where aqueous humor is drained, is damaged in myopic eyes, which increases the intraocular pressure and damages the optic nerve head [11]. In addition, one study found that highly myopic eyes tend to have a thinner lamina cribrosa due to the long axial length, which could help explain the potential relationship between highly myopic eyes and glaucoma [12]. While myopia may be a risk factor for glaucoma, a recent meta-analysis found myopia to be protective for glaucoma disease progression and subsequently decrease visual field loss. The researchers theorized that the lamina cribrosa defect from myopia may release pressure so that the risk of visual field loss is decreased [13].

Detecting meaningful changes in visual field progression is important for glaucoma management and may or may not be impacted by myopia. Visual field tests are used to test visual function in glaucoma patients and help guide treatment [14]. Global indices such as mean deviation (MD), threshold sensitivity, and visual field indices (VFIs) are used to quantify changes in visual fields. MD measures reduction in visual field sensitivity compared with age-matched controls and is presented in values deviating from 0 [15]. Threshold sensitivity values are measured by a testing algorithm and produced at each test point in units of decibels [16]. VFI measures visual field loss as a percentage, with a normal visual field having a VFI of 100% and a perimetrically blind visual field having a VFI of 0% [17].

Detection of glaucomatous progression is critical for timely interventions; therefore, it is important to differentiate between fluctuation and pathological changes in the visual field. There are broadly two different types of fluctuation in visual fields: short-term fluctuation and long-term fluctuation. Short-term fluctuation is defined as the variability between multiple visual fields performed on the same day and not due to disease progression. Long-term fluctuation is defined as the variability between different visual fields taken during different days or months where the change is not due to disease progression [18]. Clinicians should know the amount that visual field measurements are expected to vary in clinically stable patients [19]. Some degree of visual field fluctuation is expected in all patients, with more fluctuation occurring in eyes with increased severity of glaucoma damage [20]. These patient populations that commonly show visual field fluctuations should be examined carefully to prevent incorrect diagnoses so that detection of true progression is not delayed and to prevent erroneous assumptions that disease progression has occurred [21]. This study will investigate if glaucomatous eyes with high myopia (≤ −5 diopters) show greater visual field fluctuation compared with glaucomatous eyes without myopia (−2–+2 diopters).

## 2. Methods

### 2.1. Data Collection

Data were collected from at least four visual field tests from glaucoma patients with and without myopia as defined in this study using paper medical charts or Epic electronic medical record (EMR) (Epic Systems Corporation, Verona, WI) depending on location. Regenstreif Data Services, who organizes data from health systems across Indiana, generated a list of patients with glaucoma at Indiana University (IU) Health and Eskenazi Health ophthalmology clinics in Indianapolis, Indiana, to expedite the chart review. Deidentified data were recorded in a secure Excel (Microsoft Corporation, Seattle, WA) spreadsheet following the Health Insurance Portability and Accountability Act of 1996 policies on electronic protected health information. Institutional Review Board (IRB)/Ethics Committee approval was obtained from IU IRB. This study was not registered as a clinical trial. This research adhered to the tenets of the Declaration of Helsinki.

MD, threshold sensitivity, and VFI were recorded from 24–2 or 30–2 Humphrey Visual Field tests (Carl Zeiss Meditec, Dublin, CA) to examine fluctuation. Additional information that was recorded includes age, sex, race, ocular surgeries, duration between surgery and examination, antiglaucoma medications, retina nerve fiber layer thickness, best corrected visual acuity, intraocular pressure (IOP), axial length, false positives and negatives, fixation losses, and test duration. False positive and false negative rates were provided by the testing algorithms in 24–2 and 30–2 Humphrey Visual Fields.

### 2.2. Sample Characteristics

Two groups with 78 eyes each were created: (1) a control group of emmetropic glaucoma eyes with a spherical equivalent refraction between +2 and −2 diopters and (2) a group of high myopia glaucoma eyes with a spherical equivalent refraction ≤ −5 diopters. In patients whose refractive error changed over time, the emmetropic group was within the +2 or −2 diopter range through all visual field exams and the myopic group was ≤ −5 diopters for all visual field exams. Patients were tested with a lens correction placed in the Humphrey Visual Field machine. All patients included were 18 years or older and had a diagnosis of primary open-angle glaucoma (POAG) prior to the visual field examinations. Diagnosis of POAG involved identifying glaucomatous optic neuropathy based on any of the following signs: optic nerve rim thinning, notching, excavation, retinal nerve fiber layer defects, or asymmetry in the vertical cup/disc ratio of ≥ 0.2 between the two eyes with associated visual field loss.

### 2.3. Exclusion Criteria

Patients with any additional ocular diseases such as macular degeneration, retinal detachments, cataracts impacting vision according to the ophthalmologist, and more were excluded. Additionally, any ocular trauma such as blunt or stab injuries, any refractive errors outside of the inclusion criteria window, any neurological diseases such as stroke, multiple sclerosis, giant cell arteritis, and more, or systemic diseases that manifest in the eyes such as diabetic retinopathy and hypertensive retinopathy were excluded. To ensure long-term fluctuation was being measured and not short-term fluctuation, visual fields taken on the same day were excluded. Visual fields with obvious artifacts according to documentation in the patient's chart by the ophthalmologist were not used. Reliable visual field results were defined as ≤ 33% false positives, ≤ 33% false negatives, and pupil diameter ≥ 3 mm. Visual field tests with retinal sensitivity negative slope over −1.5 dB/year were excluded for visual field progression.

### 2.4. Statistical Analysis

Sample size: In the study by Rabiolo et al. [22], the median with interquartile range (IQR) for root mean square error of approximation (RMSEA) of MD was 0.97 dB (0.71–1.40 dB); standard deviation (SD) estimated from the IQR is 1.35 dB. With a sample size of 78 per group (156 total), the study has 80% power to detect a difference in MD RMSEA of 0.5 dB between the two groups, based on a two-sided 5% significance level.

Statistical methods: Fluctuation findings were summarized for the MD, threshold sensitivity, and VFI overall and by group. RMSEA two sample -tests were used to analyze fluctuation for all three measures. RMSEA values for all three outcomes were compared between the two groups using a one-way analysis of variance (ANOVA) with a random effect on subject. Bland Altman plots were plotted for individual “difference from mean” on *Y*-axis vs. “patient” number in each group on the *X*-axis by group. Plotting these data shows the within-subject variability as the individual data points for each patient. All the analysis is done at a 5% significance level. SAS 9.4 (Cary, NC) was the software used to run the analysis.

## 3. Results

**Figure 1:**
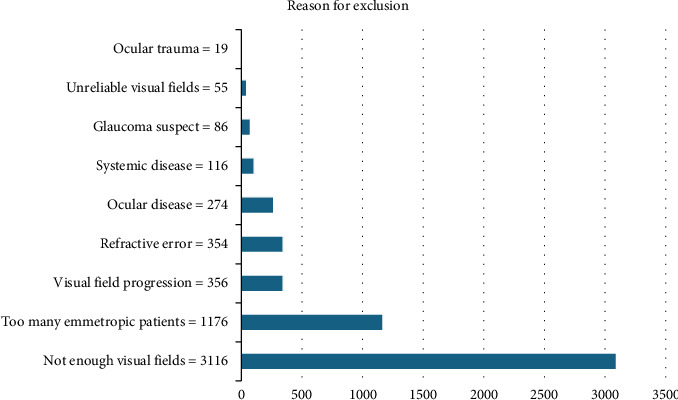
Reasons for exclusion of 5552 charts.

A total of 5655 charts were reviewed to find data for 156 eyes between June and December 2023. Data were extracted for the first chronologically identified 78 emmetropic glaucoma eyes that met inclusion criteria, and any additional emmetropic glaucoma eyes were excluded during the search for 78 high myopic eyes with glaucoma. A total of 50 patients had one eye included, and 53 patients had both eyes included. A total of 22 myopes had one eye included and 28 had both eyes included. A total of 28 emmetropes had one eye included and 25 had both eyes included. Common reasons for exclusion included patients not having enough visual fields (3116 charts, 56.1%), too many emmetropes (1176 charts, 21.2%), visual field progression (356 charts, 6.4%), spherical equivalent refraction that did not meet inclusion criteria (354 charts, 6.4%), additional ocular diseases (274 charts, 4.9%), systemic diseases that manifested in the eyes (116 charts, 2.1%), glaucoma suspects (86 charts, 1.6%), patients that did not have enough reliable visual fields (55 charts, 0.97%), and ocular trauma (19, 0.33%) (Figure 1).

Demographic information including age, sex, and race was collected in 156 patients with glaucoma. The mean age was 59.63 ± 12.70 (mean ± SD). Females comprised 61.54% of the sample. Race was distributed as White = 35.90%, Black or African American = 49.36%, Asian = 7.69%, more than one race = 0.64%, and unknown = 6.41%. Demographic information in 78 emmetropic patients with glaucoma included a mean age of 63.53 ± 12.58. Females comprised 65.38% of the sample. Race was distributed as White = 17.95%, Black or African American = 65.38%, Asian = 3.85%, more than one race = 1.28%, and unknown = 11.54%. Demographic information in 78 myopic patients included a mean age of 53.61 ± 11.68. Females comprised 57.69% of the sample. Race was distributed as White = 53.85%, Black or African American = 33.33%, Asian = 11.54%, more than one race = 0%, and unknown = 1.28% (Table 1).

The average number of visual fields in the emmetropic glaucoma group was 4.83 and myopic glaucoma group was 5.62. The average amount of time in between each visual field test was 451 ± 273 days in the emmetropic glaucoma group and 438 ± 242 days in the myopic glaucoma group. The two sample *t*-test between the two means was not significant (*p*=0.49). In the emmetropic glaucoma group, the mean for MD was −6.61 ± 5.59 with a median of −4.64 compared with the myopic glaucoma group with a mean of −5.78 ± 5.87 with a median of −3.82. The two sample *t*-test between the two means was not significant (*p*=0.365). The mean of threshold sensitivity in the emmetropic glaucoma group was 22.73 ± 5.52 and the median was 24.1. In the myopic glaucoma group, the threshold sensitivity mean was 24.4 ± 5.73 and median was 26.1. The two sample *t*-test between the means was not statistically significant (*p*=0.062). The emmetropic glaucoma group mean for VFI was 84% ± 17% with a median of 95% compared with the myopic glaucoma group with a VFI mean of 86% ± 16% and median of 93%. The two sample *t*-test between the means was not statistically significant (*p*=0.40) (Table 2).

Test–retest fluctuation in MD, threshold sensitivity, and VFI were analyzed for all 156 patients with glaucoma. For fluctuation in MD, the mean was 1.31 ± 0.87 and the median was 1.18. The fluctuation in threshold sensitivity mean was 1.47 ± 0.88 and median was 1.33. Fluctuation in VFI had a mean of 0.03 ± 0.04 and median was 0.02 (Table 3). Fluctuation in MD, threshold sensitivity, and VFI were also compared between 78 myopic and 78 emmetropic glaucoma patients. Within the emmetropic glaucoma group, the fluctuation in MD mean was 1.12 ± 0.65 and median was 1.10. The fluctuation in threshold sensitivity mean was 1.34 ± 0.69 and median was 1.25. Fluctuation in VFI mean was 0.03 ± 0.02 and median was 0.02. In myopic glaucoma patients, the fluctuation in MD mean was 1.50 ± 1.02 and median was 1.27. The fluctuation in threshold sensitivity mean was 1.61 ± 1.02 and median was 1.46, where fluctuation in VFI mean was 0.04 ± 0.05 and median was 0.03 (Table 4).

**Figure 2:**
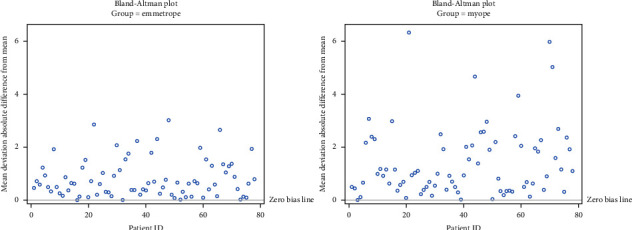
Absolute difference of means in mean deviation by group.

**Figure 3:**
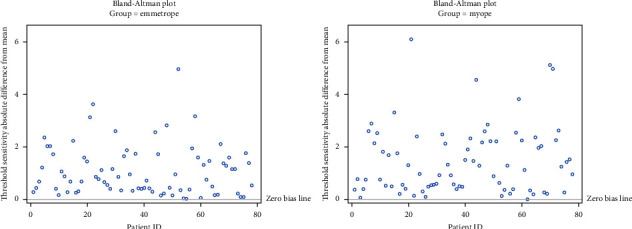
Absolute difference of means in threshold sensitivity by group.

**Figure 4:**
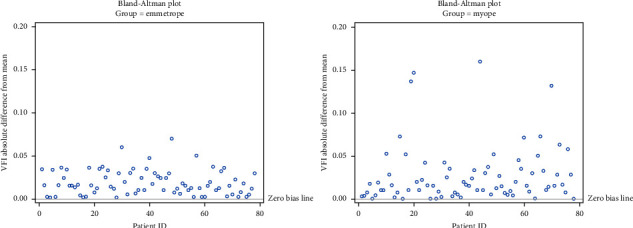
Absolute difference of means in VFI by group.

One-way ANOVA tests with random effect on subject was used to compare 78 emmetropic glaucoma patients with 78 myopic glaucoma patients using fluctuation in MD, threshold sensitivity, and VFI. For fluctuation in MD, the difference was −0.29 (*p*=0.01). Fluctuation in threshold sensitivity had a difference of −0.19 (*p*=0.11). Fluctuation in VFI had a difference of −0.30 (*p*=0.03) (Table 5). Bland Altman plots show fluctuation for MD (Figure 2), threshold sensitivity (Figure 3), and VFI (Figure 4).

RMSEA two sample *t*-tests between 78 myopic glaucoma eyes and 78 emmetropic glaucoma eyes were also used to compare fluctuation of MD, threshold sensitivity, and VFI. In the emmetropic group, fluctuation in MD results were mean equal to 1.125 ± 0.652 and the standard error of the mean (SEM) measured as 0.074 compared with the myopic group with a mean equal to 1.504 ± 1.016 and SEM measured as 0.115 (*p*=0.006). Fluctuation in threshold sensitivity measures in the emmetropic group were mean equal to 1.339 ± 0.687 and SEM measured as 0.078 compared with the myopic group of a mean equal to 1.609 ± 1.022 and SEM measured as 0.116 (*p*=0.054). Measures of fluctuation in VFI in the emmetropic group had a mean equal to 0.026 ± 0.019 and SEM measured as 0.002 compared with the myopic group mean equal to 0.037 ± 0.045 and SEM measured as 0.005 (*p*=0.035) (Table 6).

## 4. Discussion

Using parameters on visual field tests—including MD, VFI, and threshold sensitivity—we examined if myopic glaucoma patients demonstrated long-term visual field fluctuation over time. There was a statistically significant difference in MD and VFI fluctuation between myopic glaucoma groups and emmetropic glaucoma groups but not in threshold sensitivity.

Strengths of this study include multiple measures of fluctuation used, and each measure has its own benefits. MD, for example, best reflects overall change in the eye [23]. VFI is meant to eliminate the confounding variable of cataracts by disregarding any reduction in sensitivity unless it is outside of the normal range for pattern deviation. It also weighs central visual field loss more heavily than peripheral visual field loss [17]. The VFI can allow a clinician to understand what visual field changes are specific to glaucomatous damage.

Each measure of fluctuation also has its own weakness. MD is not sensitive to small, localized defects that occur in early glaucoma, and may be impacted by cataracts [24]. Likewise, VFI could underestimate the change occurring in the early stages of glaucoma when the visual field appears normal [25]. This is called the ceiling effect, where patients with little to no visual field defects and good vision may have fluctuation masked due to the high starting point [26]. Additionally, the floor effect is another weakness that can occur in eyes with large visual field defects and poor vision where disease progression can no longer be detected and the visual field appears stable [27, 28]. In this study, 11 (14%) emmetropic and 22 (28%) myopic eyes started with VFIs near the upper limits of 100% and may have demonstrated the ceiling effect. A total of 1 emmetropic and 1 myopic eye started with VFIs below 15% and may have demonstrated the floor effect. The ceiling and floor effect may mask fluctuation between visual fields. Additionally, pattern standard deviation (PSD) was not analyzed in this study. PSD reflects age-corrected differences in sensitivity and shows localized defects that occur in glaucoma [29]. Fluctuation of PSD could be analyzed in future studies.

When patients have large visual field defects, the 10–2 Humphry visual field is typically used instead of the 24–2 or 30–2 Humphrey Visual Field. The 10–2 Humphry Visual Field is better at detecting central visual field progression, which is often the last part of the visual field preserved in advanced glaucomatous damage [30]. The 10–2 Humphrey Visual Field does not provide data for VFI; therefore, another limitation to this study is that patients with poor visual acuity were disproportionately excluded due to not having enough visual fields with VFI values.

Additionally, there were differences in demographics between the emmetropic and myopic glaucoma groups. The myopic glaucoma group had a mean age of 53.61 ± 11.68, compared with the emmetropic glaucoma group with a mean age of 63.53 ± 12.58, which was statistically significant (*p* ≤ 0.01). Additionally, the emmetropic glaucoma group included 51 patients (65.38%) that identified as Black or African American, compared with the myopic glaucoma group with 26 patients (33.33%). The differences between groups could impact the results and is an additional weakness of the study.

Although this study demonstrates that myopic glaucoma patients demonstrate statistically significant visual field fluctuation, this study does have limitations and may not apply to patients on the extreme ends of the visual field spectrums. Additionally, patients with other ocular diseases or trauma were not included in this study, so clinicians should incorporate the patient's full history when looking for visual field fluctuation. The visual field test is also subjective and heavily reliant on patients' ability to perform the test which may be influenced by familiarity with the test, fatigue, attention span, and disease severity [31]. Finally, visual field defects may not correlate with the patient's functional vision or optic nerve changes, so the visual field test should accompany other aspects of the clinical presentation and not be the only thing considered [32].

Future directions of this study include comparing fluctuation using MD, PSD, and threshold sensitivity on 10–2, 24–2, and 30–2 Humphrey Visual Fields in patients with different stages of glaucoma. Future research can also be directed toward understanding the mechanisms behind this observed visual field fluctuation in myopic glaucoma patients. Other areas of future research could include comparing visual field fluctuation between groups with different severity of myopia to see if there is a linear relationship between fluctuation and degree of myopia. Axial length could also be analyzed for influence on fluctuation. Finally, guided progression analysis (GPA) could be used to analyze areas of the visual field that demonstrate fluctuation more than others.

## 5. Conclusion

The results for MD and VFI are consistent with the hypothesis that myopic patients demonstrate more visual field fluctuation than emmetropic glaucoma patients. Therefore, clinicians should use caution when interpreting the visual field in highly myopic eyes and consider fluctuation versus true disease progression. Physicians should incorporate more frequent visual field tests and a thorough examination of other clinical findings such as optic nerve appearance, retinal nerve fiber layer thickness, intraocular pressure, and a patient's own assessment of their vision to guide clinical decision making for myopic glaucoma patients.

## Figures and Tables

**Table 1:** Demographic information in 78 emmetropic glaucoma patients and 78 myopic glaucoma patients.

	Emmetrope (*n* = 78)	Myope (*n* = 78)	Total (*n* = 156)
*Age (years)*			
Mean (SD)	63.53 (12.58)	53.61 (11.68)^∗^	59.63 (12.70)
Median [Min, Max]	64.29 [20, 82]	55.50 [23, 80]	60.00 [20, 82]

*Sex*			
Female	51 (65.38%)	45 (57.69%)	96 (61.54%)
Male	27 (34.62%)	33 (42.31%)	60 (38.46%)

*Race*			
White	14 (17.95%)	42 (53.85%)	56 (35.90%)
Black or African American	51 (65.38%)	26 (33.33%)	77 (49.36%)
Asian	3 (3.85%)	9 (11.54%)	12 (7.69%)
More than one race	1 (1.28%)	0 (0%)	1 (0.64%)
Unknown	9 (11.54%)	1 (1.28%)	10 (6.41%)

^∗^Reaches statistical significance (*p* value < 0.05).

**Table 2:** Descriptive statistics of mean deviation, threshold sensitivity, and visual field index from 78 emmetropic glaucoma patients and 78 myopic glaucoma patients.

Variable	Group	*N*	Mean	Std. Dev	Median	Lower quartile	Upper quartile	Minimum	Maximum	*p* value
Mean deviation	Emmetrope	78	−6.61	5.59	−4.64	−7.85	−3.57	−28.49	0.17	0.365
	Myope	78	−5.78	5.87	−3.82	−6.80	−2.17	−27.99	1.39	

Threshold sensitivity	Emmetrope	78	22.73	5.52	24.1	21.1	26.3	2.76	32.5	0.062
	Myope	78	24.4	5.73	26.1	23.2	28.1	2.98	32.1	

Visual field index	Emmetrope	78	84%	17%	95%	78%	95%	17%	99%	0.40
	Myope	78	86%	16%	93%	80%	96%	16%	99%	

**Table 3:** Descriptive statistics of intertest fluctuation of mean deviation, threshold sensitivity, and visual field index from the visual field tests of 156 patients with glaucoma.

Variable	*N*	Mean	Std. Dev	Median	Lower quartile	Upper quartile	Minimum	Maximum
Mean deviation	156	1.31	0.87	1.18	0.77	1.63	0.24	6.70
Threshold sensitivity	156	1.47	0.88	1.33	0.84	1.95	0.18	6.08
Visual field index	156	0.03	0.04	0.02	0.01	0.04	0.00	0.29

**Table 4:** Descriptive statistics of intertest fluctuation of mean deviation, threshold sensitivity, and visual field index from the visual field tests of 78 emmetropic glaucoma patients and 78 myopic glaucoma patients.

Group	Variable	*N*	Mean	Std. Dev	Median	Lower quartile	Upper quartile	Minimum	Maximum
Emmetrope	Mean deviation	78	1.12	0.65	1.10	0.73	1.40	0.24	4.84
	Threshold sensitivity	78	1.34	0.69	1.25	0.79	1.76	0.28	3.48
	Visual field index	78	0.03	0.02	0.02	0.01	0.03	0.00	0.11

Myope	Mean deviation	78	1.50	1.02	1.27	0.87	1.88	0.34	6.70
	Threshold sensitivity	78	1.61	1.02	1.46	0.94	2.12	0.18	6.08
	Visual field index	78	0.04	0.05	0.03	0.02	0.04	0.00	0.29

**Table 5:** One-way ANOVA tests of mean deviation, threshold sensitivity, and visual field index in 78 emmetropic glaucoma patients compared with 78 myopic glaucoma patients.

Variable	Effect	Group	Versus	Group	Difference	*p* value
Mean deviation	Group	Emmetrope	<	Myope	−0.29	0.01^∗^
Threshold sensitivity	Group	Emmetrope	NSD	Myope	−0.19	0.11
Visual field index	Group	Emmetrope	<	Myope	−0.30	0.03^∗^

^∗^Reaches statistical significance (*p* value < 0.05).

**Table 6:** Two sample *t*-test of RMSEA values of intertest fluctuation of mean deviation, threshold sensitivity, and visual field index from the visual field tests of 78 emmetropic glaucoma patients and 78 myopic glaucoma patients.

Variable	Group	Mean	Standard deviation	Standard error of the mean	*p* value
Mean deviation	Emmetrope	1.125	0.652	0.074	0.006^∗^
	Myope	1.504	1.016	0.115	

Threshold sensitivity	Emmetrope	1.339	0.687	0.078	0.054
	Myope	1.609	1.022	0.116	

Visual field index	Emmetrope	0.026	0.019	0.002	0.035^∗^
	Myope	0.037	0.045	0.005	

^∗^Reaches statistical significance (*p* value < 0.05).

## Data Availability

The data that support the findings of this study are available from the corresponding author upon reasonable request.
